# Distinct Modulation of Feeding Behavior in the Whitefly Vector *Bemisia tabaci* MED by ToCV Single-Infection Versus Synergistic Co-Infection with TYLCV

**DOI:** 10.3390/insects16111091

**Published:** 2025-10-24

**Authors:** Tianbo Ding, Hong Huang, Xiaobei Liu, Min Zhang, Jianmei Yu, Guoxu Xia, Dong Chu

**Affiliations:** 1 Shandong Engineering Research Center for Environment-Friendly Agricultural Pest Management, College of Plant Health and Medicine, Qingdao Agricultural University, Qingdao 266109, China; hhong0809@163.com (H.H.); xiaobeiliu7@163.com (X.L.); zhangminqau@163.com (M.Z.); cauyjm@163.com (J.Y.); xiaguoxuqau@163.com (G.X.); 2 Zibo Academy of Agricultural Sciences, Zibo 255033, China

**Keywords:** *Bemisia tabaci* MED, co-infection, feeding behavior, performance, viral epidemic

## Abstract

Plant viruses can significantly influence the behavior and performance of their insect vectors, with profound implications for viral epidemiology. However, studies on the effects of co-infection with multiple plant viruses on vector feeding behavior remain scarce, despite its frequent occurrence in nature and potential for altered transmission outcomes. *Bemisia tabaci* MED, a key vector insect, is closely linked to the rapid spread of tomato chlorosis virus (ToCV) and tomato yellow leaf curl virus (TYLCV) in China. In this study, the electrical penetration graph (EPG) technique was employed to investigate and compare the indirect (via infected plants) and direct (via viruliferous insects) effects of ToCV alone and ToCV and TYLCV co-infection on the feeding behaviors of *B. tabaci* MED. The results revealed that whiteflies on ToCV-infected or ToCV&TYLCV co-infected plants exhibited significantly longer non-probing durations compared to those on un-infected plants. The intracellular puncture activity of whiteflies was markedly reduced on virus-infected plants, and ToCV infection particularly shortened the duration of phloem sap ingestion. Moreover, viruliferous whiteflies (carrying ToCV or both viruses) spent less time in the intercellular pathway phase. Specifically, ToCV-viruliferous whiteflies had a shorter first-probe duration than non-viruliferous ones. The time from the first probe to the first E phase was also shorter in viruliferous whiteflies, especially in those carrying both ToCV and TYLCV. Furthermore, a significant difference was observed in the total duration of phloem sap ingestion between ToCV-viruliferous and ToCV&TYLCV-viruliferous whiteflies. These findings indicate that both ToCV infection and ToCV&TYLCV co-infection can modulate whitefly feeding behaviors through indirect and direct manners, with co-infection eliciting unique behavioral changes. These insights are valuable for elucidating the negative impact of ToCV-infected and ToCV&TYLCV co-infected tomato plants on whitefly performance, and for uncovering the mechanisms underlying the epidemics of these viruses.

## Simple Summary

Plant viruses can alter insect feeding behavior, which in turn affects disease transmission dynamics. However, the effects of viral co-infections, a common scenario in field conditions, on these behaviors are poorly understood. This study investigated how two widespread tomato viruses—tomato chlorosis virus (ToCV) alone or in co-infection with tomato yellow leaf curl virus (TYLCV)—influence the feeding behavior of whiteflies, the key insect vector responsible for their spread in China. Using electrical penetration graph (EPG) technology, we analyzed plant—insect interactions between infected tomato plants and viruliferous whiteflies. The results showed that whiteflies on virus-infected plants exhibited impaired feeding patterns, characterized by increased inactivity and reduced phloem sap ingestion. Viruliferous whiteflies (carrying ToCV or both viruses) initiated feeding more rapidly but consumed less sap overall. Notably, the feeding behavior of whiteflies carrying both viruses was distinct from that of those carrying only one virus. These findings elucidate the mechanisms underlying the rapid spread of these virus complexes and suggest how infected plants might mount a natural defense against whitefly damage. By revealing how viruses manipulate insect behavior, this research provides insights for developing targeted strategies to manage viral outbreaks in agriculture.

## 1. Introduction

The sweet potato whitefly, *Bemisia tabaci* (Gennadius) (Hemiptera: Aleyrodidae) is a species complex consisting of at least 44 cryptic species [1] and has brought severe damage to vegetable industry worldwide [2,3]. As an important vector insect, *B. tabaci* can transmit more than 200 plant viruses, which belongs to five genera (*Begomovirus*, *Crinivirus*, *Torradovirus*, *Ipomovirus*, and *Crinivirus*) [4,5,6]. In China, the *B. tabaci* MED, one member of the species complex, has gradually become the dominant cryptic species over the past decades [7]. As a vector whitefly, *B. tabaci* MED was considered to be closely associated with the epidemics of tomato yellow leaf curl virus (TYLCV) and tomato chlorosis virus (ToCV) in China [8,9,10].

Following the introduction of TYLCV into China’s mainland in 2006, another plant virus, ToCV, was found in China’s mainland in 2012 [7]. ToCV, a member of Crinivirus, was first reported in northern Florida, United States [11], and has rapidly spread into South America, Africa, Europe, and Asia [12,13,14,15]. As a newly emerged virus, ToCV has shown a devastating effect on cultivated vegetable production including tomato. Owing to the common vector, ToCV&TYLCV co-infection has been reported in several provinces of China, including Shandong, Yunnan, and Jiangsu [16,17,18]. For the *B. tabaci* MED vector, TYLCV is a persistently transmitted plant virus, while ToCV is a semipersistently transmitted plant virus. However, despite advances in understanding how TYLCV or ToCV alone manipulate the *B. tabaci* MED vector, the mechanisms by which their co-infection alters vector behavior (e.g., feeding, host selection) and influences the epidemic dynamics of both viruses remain poorly understood.

The research on the interaction between the virus-vector insect and host plant, especially under co-infection scenarios, will be helpful to reveal the mechanism of the epidemics of these viruses and their management [19,20,21,22]. The majority of plant viruses are transmitted by vector insects, most of which have piercing–sucking mouthparts [23]. The modification of feeding behavior caused by virus infection is considered the crucial strategy for viral transmission [24,25,26,27]. For example, TSWV-infected male western flower thrips, *Frankliniella occidentalis*, exhibited significantly elevated feeding activity compared to their un-infected counterparts, demonstrating up to a threefold increase in all feeding behaviors, resulting in heightened viral inoculation efficacy [24]. Accumulating evidence has pointed out that numerous plant viruses can directly (i.e., by infecting and acting within the vector inset itself) or indirectly (i.e., by altering the host plants, which subsequently affects the vector) alter the performance and behaviors of the vector insects, exhibiting an ecological implication for the virus epidemic [28,29,30]. Until now, most studies have focused on single-virus manipulation, leaving a significant gap in knowledge regarding the effects of co-infection of multiple plant viruses, such as ToCV and TYLCV, on the behavior of vectors.

TYLCV infection indirectly improved the performances of *B. tabaci* MED on its hosts, such as of survival and reproduction [19,20]. Furthermore, TYLCV can also directly modulate the behavior of its whitefly vector to facilitate transmission. Previous researchers found that TYLCV-viruliferous *B. tabaci* MED could feed more often and exhibited a longer period for salivating into phloem sieve elements, leading to enhanced transmission ability [25]. However, the observed reduction in probing attempts on ToCV-infected plants suggests that the virus may disrupt vector physiology and behavior through a plant-mediated, indirect mechanism [31]. Ontiveros et al. [32] conducted a comparative evaluation of the effects of single infections (ToCV or TYLCV) versus co-infection (ToCV&TYLCV) on host selection preference in whitefly vectors. It was found that tomato plants infected with TYLCV alone or co-infected with TYLCV and ToCV exhibited significantly greater attractiveness to whiteflies (*B. tabaci*) compared to plants infected solely with ToCV [32]. To date, few studies have systematically investigated the impacts of ToCV infection and its co-infection with TYLCV on the feeding behavior of whitefly vectors (*B. tabaci* MED).

In the present study, we aimed to evaluate and compare the indirect and direct effects of ToCV and ToCV&TYLCV on feeding behavior of *B. tabaci* MED using the electrical penetration graph (EPG) technique. Thus, two experiments were conducted: (1) record the feeding behavior of *B. tabaci* MED on ToCV-infected, ToCV&TYLCV co-infected, and healthy tomato plants; (2) record the feeding behavior of ToCV-viruliferous, ToCV&TYLCV-viruliferous, and non-viruliferous *B. tabaci* MED on cotton plants. The results may help enlarge our knowledge about the manipulations on vector insects caused by diverse forms of viral infection and provide further understanding on the epidemiology of plant viruses transmitted by insects.

## 2. Materials and Methods

### 2.1. Plants, Whitefly Population, and Viruses

Cotton plants (*Gossypium hirsutum* L. cv. Lu-Mian 21) and tomato plants (*Solanum lycopersicum* M. cv. Zhongza 9) were cultivated in square flowerpots (6.5 cm × 6.5 cm × 6.7 cm) in a climate chamber at a 16:8 (L:D) photoperiod, 27 ± 1 °C, and 60 ± 5% RH. Cotton and tomato plants at the 2–3-true-leaf stage were used in the experiments.

The population of *B. tabaci* MED was reared on cotton plants, which are immune to both ToCV and TYLCV for nearly ten years under the conditions above. The identity of the whitefly MED population was periodically ensured using the *Vsp I*-based *mtCOI*-RFLP method [33]. Genomic DNA was extracted from individual adult whiteflies using the TIANamp Micro DNA Kit (TIANGEN, Beijing, China). These DNA samples served as templates to amplify an approximately 620 bp fragment of the *mtCOI* gene via PCR with specific primers (Supplementary Table S1) [34]. Amplifications were performed using a PCR Thermal Cycler Dice Model TP600 (TaKaRa, Kusatsu, Japan). Each 25 μL reaction mixture consisted of 12.5 μL of 2 × Accurate Taq Master Mix (dye plus) II (ACCURATE BIOLOGY, Changsha, China), 1 μL of each forward and reverse primer, 1 μL of DNA template, and 9.5 μL of ddH_2_O. The thermal cycling protocol comprised an initial denaturation at 94 °C for 30 s; 35 cycles of denaturation at 98 °C for 10 s, annealing at 52 °C for 30 s, and extension at 72 °C for 1 min; followed by a final extension at 72 °C for 2 min. Subsequently, 20 μL of each PCR product was digested with the restriction enzyme *Vsp I* (New England Biolabs, Ipswich, MA, USA). The digested products were separated by electrophoresis on 1.0% agarose gel, and the biotypes (MED or MEAM1) were determined based on the number and size of the restriction fragments [34,35]. The ToCV-infected and ToCV&TYLCV co-infected tomatoes were found and collected from Qingdao, Shandong Province, China, and the virus isolates were maintained in “Zhong za 9” tomato plants by *B. tabaci*-mediated transmission. The purity of the virus isolates maintained in the laboratory was regularly tested by PCR [36,37].

### 2.2. Establishment of ToCV-Infected and ToCV&TYLCV Co-Infected Tomato Plants

A total of 25 male *B. tabaci* MED adults were collected and placed on ToCV-infected and ToCV&TYLCV co-infected tomato plants for an acquisition access period (AAP) of 48 h. Then, the whiteflies were transferred into a clip cage attached on the second true leaf (from bottom to top) of the healthy 2–3-true-leaf-stage test tomato plants for an inoculation access period (IAP) of 48 h. Similarly, the un-infected tomato plants were generated by male whiteflies through a 48 h AAP on healthy tomatoes and a 48 h IAP on test tomato plants. Fifty tomato plants were prepared for each treatment. When the virus-infected and un-infected tomato plants reached the 4–5-true-leaf stage (30 days after inoculation), they would be used for EPG experiments. We confirm the virus infection through PCR [36,37,38] after EPG recording, in order to avoid the influence on the probing activities of *B. tabaci* brought by physical injury. The total RNA of the tomato plants (ToCV-infected, ToCV&TYLCV co-infected, and healthy) was extracted using TRIzol Reagent (Thermo Fisher, Waltham, MA, USA). First-strand cDNA was generated following the manufacturer’s instructions of the Evo *M-MLV* RT Mix Kit with gDNA Clean for qPCR Ver. 2 (Accurate, Changsha, China). The DNA of the tomato plants was extracted by means of a Plant Genomic DNA Kit (TIANGEN, Beijing, China) according to the manufacturer’s protocol. Reactions were performed in a 13 μL mixture containing 11 μL of Golden Star T6 super PCR Mix (TsingKe, Beijing, China), 0.5 μL of each primer (Supplementary Table S1) [36,37], and 1 μL of cDNA or DNA. The thermal cycling program consisted of the following: initial denaturation at 98 °C for 2 min; 35 cycles of 98 °C for 10 s (denaturation), 60 °C for 15 s (annealing), and 72 °C for 15 s (extension); followed by a final extension at 72 °C for 5 min.

### 2.3. Establishment of ToCV-Viruliferous and ToCV&TYLCV-Viruliferous Whitefly Colonies

A total of 300 newly emerged female whiteflies (24 h old) from the laboratory populations were collected and transferred onto ToCV-infected, TYLCV&ToCV co-infected, and un-infected tomato plants for a 24 h AAP, respectively. We employed a detection method analogous to that described above, randomly selecting 60 adult whiteflies from each treatment group. Half of these (n = 30) were allocated to individual screening for ToCV presence, while the remaining 30 were subjected to individual TYLCV detection. Other viruliferous and non-viruliferous whiteflies were used for EPG experiments.

### 2.4. Electrical Penetration Graph (EPG) Recording

Two experiments were designed and conducted: (1) measurement of the feeding behavior of *B. tabaci* MED on ToCV-infected, ToCV&TYLCV co-infected, and un-infected tomato plants; (2) measurement of the feeding behavior of ToCV-viruliferous, ToCV&TYLCV-viruliferous, and non-viruliferous *B. tabaci* MED on cotton plants. The feeding behaviors of whitefly females during 6 h periods were monitored and recorded using a direct-current EPG (DC-EPG, Giga-8) system (Wageningen University, Wageningen, The Netherlands). *B. tabaci* adults were immobilized through an ice bath of 5 min, and a gold wire (2.0 cm × 12.5 μm) was attached to the whitefly pronotum using a small drop of water-based silver glue. After an acclimation and starvation period of 30 min, the wired whiteflies were placed on the lower surface of the second true leaf (from top to bottom) of test tomato (4–5-true-leaf stage) or cotton (2–3-true-leaf stage) plants, and connected to the EPG system. Each new whitefly and each fresh plant were considered one replicate, which were used only once. The experiments were conducted in a room at 27 ± 1 °C and 60 ± 5% RH inside a Faraday cage. The digitized EPG signals were stored and analyzed with Stylet+ for Windows software (https://www.epgsystems.eu, 16 April 2018) (Wageningen University, The Netherlands).

### 2.5. Data Analysis

The EPG waveforms previously described for *B. tabaci* MED [25,29,39] were categorized as follows: NP (non-probing), C (pathway), pd (potential drop), E1 (phloem salivation), and E2 (phloem sap ingestion). In total, 20 EPG variables, including 12 non-phloem phase parameters and 8 phloem phase parameters, were chosen for the analysis of the two experiments. We carried out all statistical analyses using SPSS 23.0 (IBM, Armonk, NY, USA) at a 0.05 significance level. Whether the data conform to normal distribution was checked at the beginning. The data following a normal distribution were analyzed using Tukey’s-b test (one-way ANOVA), whereas a Kruskal–Wallis H test was performed if the data did not fit a normal distribution.

## 3. Results

We performed EPG analyses on non-viruliferous *B. tabaci* MED feeding on viruses-infected and un-infected tomato plants, as well as viruliferous and non-viruliferous *B. tabaci* MED feeding on cotton plants. A total of 146 successful EPG recordings were acquired, including 72 for non-viruliferous whiteflies on tomato plants (24 replicates for ToCV-infected plants, 24 replicates for ToCV&TYLCV co-infected plants, and 24 replicates for un-infected plants), and 74 for viruliferous/non-viruliferous whiteflies on cotton plants (25 replicates for ToCV-viruliferous whiteflies, 24 replicates for ToCV&TYLCV-viruliferous whiteflies, and 25 replicates for non-viruliferous whiteflies).

### 3.1. Feeding Behavior at Non-Phloem Phase of Bemisia tabaci on ToCV-Infected, ToCV&TYLCV Co-Infected, and Un-Infected Tomato Plants

**Figure 1:**
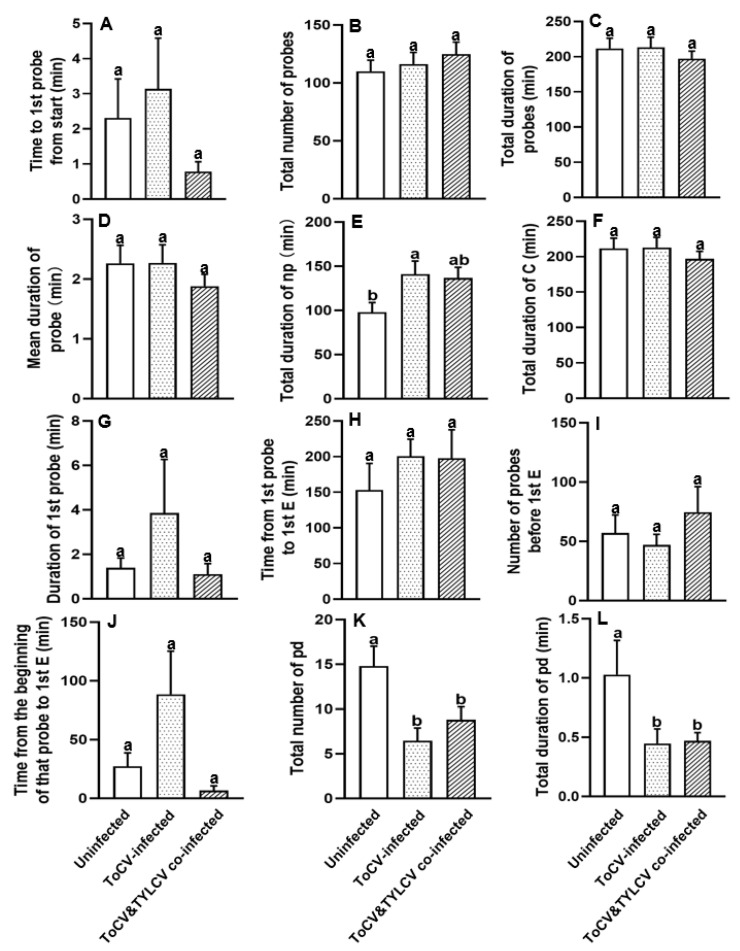
Non-phloem parameters (**A**–**L**) of *Bemisia tabaci* MED feeding on ToCV-infected, ToCV&TYLCV co-infected, and un-infected tomato plants. Np: non-probe activity; C: intercellular stylet pathway; pd: short intracellular punctures; E: phloem-related activities [40,41]. The columns and bars indicate the mean and the standard error of the mean for each parameter, respectively. Different letters above the bars represent significant differences among three groups (*p* ≤ 0.05). White bars, whiteflies feeding on un-infected plants; white dotted bars, whiteflies feeding on ToCV-infected plants; white striped bars, whiteflies feeding on TYLCV&ToCV co-infected plants.

Whiteflies had significantly fewer potential drops on both ToCV-infected (6.45 ± 1.44) and ToCV&TYLCV co-infected tomato plants (8.80 ± 1.45) than those on un-infected plants (14.80 ± 2.22) (*F*_2, 28_ = 4.374, *p* < 0.05; Figure 1K, Table 1). In addition, the total duration of potential drops was significantly shorter on viruses-infected tomato plants (0.45 ± 0.12 min for ToCV infection; 0.47 ± 0.07 min for ToCV&TYLCV co-infection) than on un-infected plants (1.03 ± 0.29 min) (*F*_2, 28_ = 4.353, *p* < 0.05; Figure 1L, Table 1). Compared to the feeding on un-infected tomato plants, *B. tabaci* took longer for non-probing when feeding on viruses-infected tomato plants (Figure 1E; Table 1). A significant difference was observed in the duration of non-probing between feeding on un-infected and ToCV-infected plants (*F*_2, 69_ = 0.035, *p* < 0.05; Figure 1E, Table 1). *B. tabaci* took more time from the first probe to the phloem phase on viruses infected plants than on un-infected plants, although there were no significant differences (*H*_2, 72_ = 5.302, *p* > 0.05; Figure 1H, Table 1).

### 3.2. Feeding Behavior at Phloem Phase of Bemisia tabaci on ToCV-Infected, ToCV&TYLCV Co-Infected, and Un-Infected Tomato Plants

**Figure 2:**
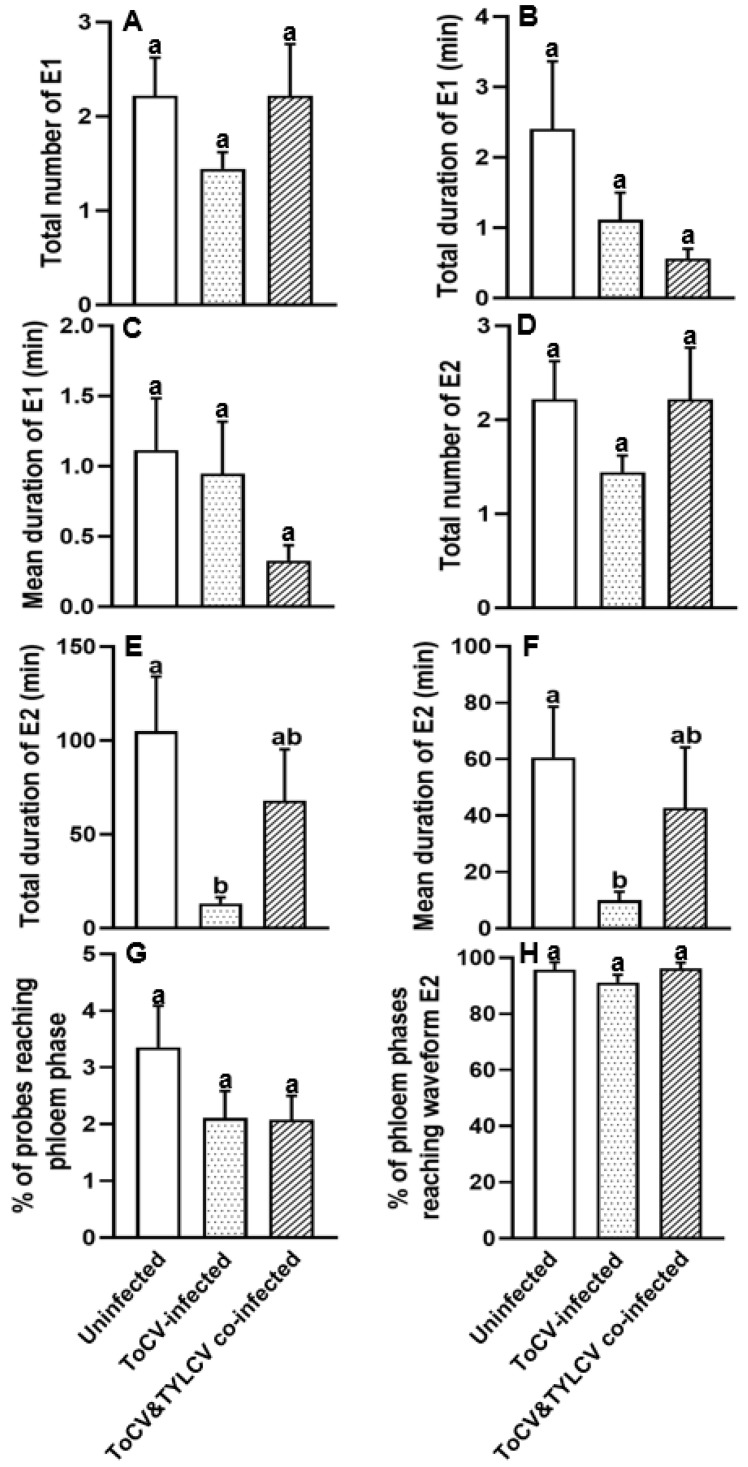
Phloem parameters (**A**–**H**) of *Bemisia tabaci* MED feeding on ToCV-infected, ToCV&TYLCV co-infected, and un-infected tomato plants. E1: salivation into a sieve element; E2: ingestion of sieve element sap [40,41]. The columns and bars indicate the mean and the standard error of the mean for each parameter, respectively. Different letters above the bars represent significant differences among three groups (*p* ≤ 0.05). White bars, whiteflies feeding on un-infected plants; white dotted bars, whiteflies feeding on ToCV-infected plants; white striped bars, whiteflies feeding on TYLCV&ToCV co-infected plants.

The effect of feeding on ToCV-infected and ToCV&TYLCV co-infected plants on the phloem feeding behaviors of whiteflies is shown in Figure 2. Although no significant differences were observed in the “total duration of E1” and “mean duration of E1”, the duration of both variables was shorter for whiteflies fed on virus-infected tomato plants than on un-infected plants (*p* > 0.05 for both comparisons; Figure 2B,C, Table 1). However, whiteflies took less phloem sap ingestions on both ToCV-infected and ToCV&TYLCV co-infected plants versus on un-infected plants (Figure 2E,F, Table 1). Furthermore, the total and mean duration of E2 were significantly decreased for whiteflies feeding on ToCV-infected tomato plants (*p* < 0.05 for both comparisons; Figure 2E,F, Table 1).

### 3.3. Feeding Behavior at Non-Phloem Phase of ToCV-Viruliferous, ToCV&TYLCV-Viruliferous, and Non-Viruliferous Bemisia tabaci on Cotton Plants

**Figure 3:**
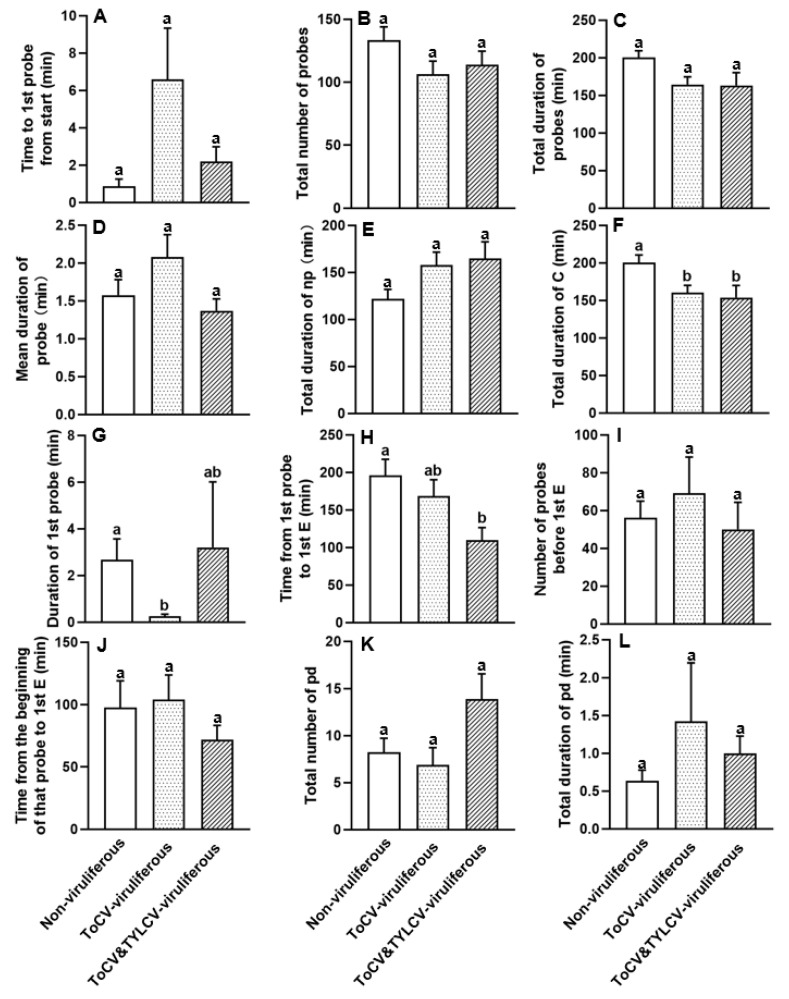
Non-phloem parameters (**A**–**L**) of ToCV-viruliferous, ToCV&TYLCV-viruliferous, and non-viruliferous *Bemisia tabaci* MED feeding on cotton plants. Np: non-probe activity; C: intercellular stylet pathway; pd: short intracellular punctures; E: phloem-related activities [40,41]. The columns and bars indicate the mean and the standard error of the mean for each parameter, respectively. Different letters above the bars represent significant differences among three groups (*p* ≤ 0.05). White bars, non-viruliferous whiteflies feeding on cotton plants; white dotted bars, ToCV-viruliferous whiteflies feeding on cotton plants; white striped bars, ToCV&TYLCV-viruliferous whiteflies feeding on cotton plants.

The duration of the intercellular style pathway “C” was significantly shorter in both the ToCV-viruliferous and ToCV&TYLCV-viruliferous cases than in non-viruliferous whiteflies (*F*_2, 71_ = 4.395, *p* < 0.05; Figure 3F, Table 1). ToCV-viruliferous *B. tabaci* had the shorter duration for the first probe on cotton plants than non-viruliferous whiteflies, while the difference was significant (*H*_2_ = 11.631, *p* < 0.05; Figure 3G, Table 1). The duration from the first probe to the phloem was shorter in viruliferous (168.38 ± 21.99 min for ToCV-viruliferous; 109.69 ± 16.90 min for ToCV&TYLCV-viruliferous) than in non-viruliferous (196.10 ± 21.28 min) whiteflies; a significant difference was observed between ToCV&TYLCV-viruliferous and non-viruliferous whiteflies (*F*_2, 28_ = 4.985, *p* < 0.05; Figure 3H, Table 1). Additionally, viruliferous whiteflies took less time and fewer probes than non-viruliferous whiteflies, although no significant differences were found (*p* > 0.05 for both comparisons; Figure 3B,C, Table 1).

### 3.4. Feeding Behavior at Phloem Phase of ToCV-Viruliferous, ToCV&TYLCV-Viruliferous, and Non-Viruliferous Bemisia tabaci on Cotton Plants

**Figure 4:**
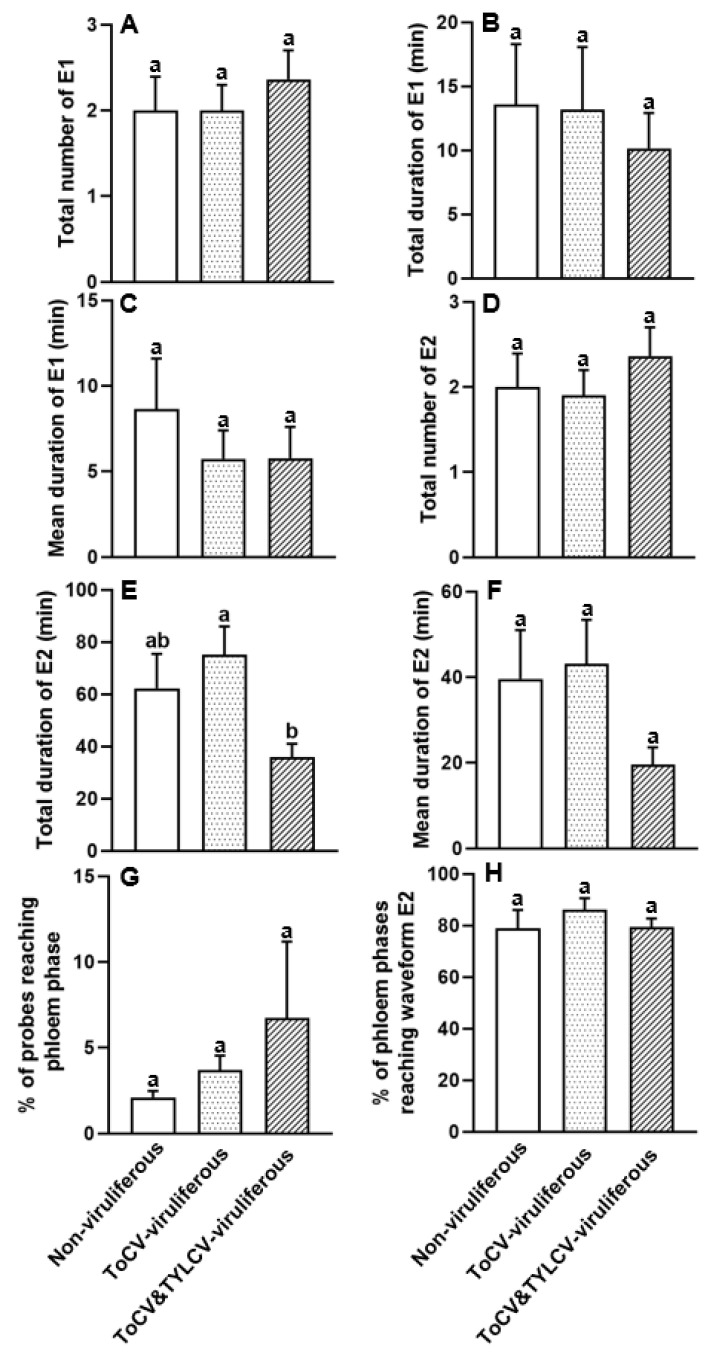
Phloem parameters (**A**–**H**) of ToCV-viruliferous, ToCV&TYLCV-viruliferous, and non-viruliferous *Bemisia tabaci* MED feeding on cotton plants. E1: salivation into a sieve element; E2: ingestion of sieve element sap [40,41]. The columns and bars indicate the mean and the standard error of the mean for each parameter, respectively. Different letters above the bars represent significant differences among three groups (*p* ≤ 0.05). White bars, non-viruliferous whiteflies feeding on cotton plants; white dotted bars, ToCV-viruliferous whiteflies feeding on cotton plants; white striped bars, ToCV&TYLCV-viruliferous whiteflies feeding on cotton plants.

The total duration of E2 was 2.09 and 1.73 times shorter in ToCV&TYLCV-viruliferous than in ToCV-viruliferous and non-viruliferous whiteflies, respectively, while a significant difference was observed between ToCV&TYLCV- and ToCV-viruliferous whiteflies (*F*_2, 28_ = 4.096, *p* < 0.05; Figure 4E, Table 1). No significant differences were found in other phloem variables including the total number of E1, total duration of E1, mean duration of E1, total number of E2, mean duration of E2, percentage of probes reaching the phloem phase, and the percentage of phloem phases reaching waveform E2, among ToCV-viruliferous, ToCV&TYLCV-viruliferous, and non-viruliferous *B. tabaci* (*p* > 0.05 for these six comparisons; Figure 4A–D,F–H, Table 1).

## 4. Discussion

The present study found that both ToCV infection and ToCV&TYLCV co-infection could indirectly modify *B. tabaci* MED feeding behavior. For instance, the total durations of non-probing were obviously longer on viruses-infected plants than on healthy plants. This finding is consistent with a previous report showing that *Aphis fabae* exhibited a prolonged non-probing period on plants infected with bean common mosaic virus (BCMV) [26], a non-persistently transmitted virus. Moreover, the first probe duration was shortened on ToCV&TYLCV co-infected plants compared to healthy controls. A similar shortening of the first probe was observed in *B. tabaci* MEAM1 feeding on plants infected with cucurbit chlorotic yellows virus (CCYV) [42], which, like ToCV, is semipersistently transmitted.

The biological significance of these alterations may be linked to the transmission modes of the viruses involved. Semipersistently transmitted viruses (e.g., ToCV, CCYV) and non-persistently transmitted viruses (e.g., BCMV) are retained by their vectors for relatively short periods. It has been hypothesized that virus-induced host changes that deter prolonged feeding or promote dispersal (e.g., increased non-probing time, shortened first probes, and deterred settling) could be advantageous for these viruses by encouraging vectors to move away from the infected plant after acquisition, thereby enhancing viral spread to new healthy hosts [42,43]. This aligns with our results showing that *B. tabaci* MED prefers un-infected plants over infected ones, with co-infected plants being the least preferred [22].

Additionally, the present study revealed that virus-infected tomato plants significantly reduced the total number and duration of potential drops. The total number of potential drops was also decreased on CCYV-infected plants compared to healthy ones [42]. Collectively, the alterations in non-probing, first probe, and intracellular puncture activities suggest that ToCV infection makes the host plant less palatable or suitable for *B. tabaci* MED.

Interestingly, the negative impact on the vector’s feeding behavior appears to be modulated by viral co-infection. While ToCV single-infection significantly reduced phloem sap ingestion, this effect was absent in ToCV&TYLCV co-infected plants. This mitigation of negative effects could be attributed to the influence of the persistently transmitted virus TYLCV. Persistently transmitted viruses may evolve strategies to enhance vector feeding or suppress plant defenses that deter vectors [29,44]. According to research, upon infecting the host plant, begomoviruses can interact with MYC2 in tomatoes, suppressing the activation of MYC2-regulated terpene synthase genes, thereby compromising resistance against whiteflies [45]. We speculate that during co-infection, TYLCV may alter the physiological or biochemical profile of the plant, effectively counteracting the defense responses triggered by ToCV and creating a more favorable environment for the whitefly. This interplay between viruses with different transmission modes has significant epidemiological implications, as it could influence the colonization patterns of vectors and the spatiotemporal dynamics of virus spread in the field.

The mechanism underlying the indirect effect of ToCV on the feeding behavior may be associated with the physiological changes in host plants caused by the infection of this virus. Previous studies found that viral infection could induce changes in host plant volatiles, which might lead to an attractive or repellent effect on vector insects [46,47]. For example, the amounts of several terpenes that might play important roles against whitefly feeding [48] were higher in ToCV-infected plants than mock-inoculated plants [47]. Additionally, the comparative transcriptome analysis revealed that numerous up-regulated genes of tomato plants in response to ToCV&TYLCV co-infection were enriched in GO terms of secondary metabolic process, defense response, and innate immune responses [49].

Phloem sap ingestion is another crucial activity of feeding behavior in phloem phase, which is considered the host plant’s acceptance by insects [50]. For example, *A. fabae* had a shorter total and mean duration of E2 feeding on cucumber mosaic virus (CMV)-infected plants compared with these on healthy plants [26]. The present study found that the total and mean duration of phloem sap ingestion in whiteflies decreased significantly on ToCV-infected tomato plants, indicating that plants infected with ToCV were less suitable than healthy plants for whiteflies feeding and surviving. Our observation is consistent with early studies that ToCV-infection could decrease the performance of *B. tabaci* MED on tomatoes [22,39,51]. However, the present study also found that ToCV&TYLCV co-infection has no significant effects on the measurements.

The present study also found that both ToCV and ToCV&TYLCV could directly modify *B. tabaci* MED feeding behavior. Our study found that both ToCV-viruliferous and ToCV&TYLCV-viruliferous whiteflies spent less time on the intercellular pathway phase, suggesting that viruliferous whiteflies do not like to conduct probe activity during non-phloem phase. Interestingly, compared with the non-viruliferous *B. tabaci* MED, the time from the first probe to the first E of ToCV&TYLCV-viruliferous counterparts was significantly reduced, which suggests that the feeding behavior of whiteflies can be manipulated by ToCV&TYLCV and benefit the transmission of these viruses. Consistent with our findings in the present study, *B. tabaci* was also found to reach the phloem of plants faster after the acquisition of CCYV or TYLCV [29,52]. Phloem salivation by whiteflies is an absolute prerequisite for the transmission of both ToCV and TYLCV, while a longer duration of the salivation phase can increase the inoculation rates of viruses [39,41]. But the present study found that there were no significant differences in the total or mean durations of E1 between viruliferous and non-viruliferous whiteflies. In addition, phloem ingestion is also important in virus transmission by vector insects. After the acquisition of potato virus Y (PVY), the cannabis aphid (*Phorodon cannabis*) spent less time ingesting phloem than non-viruliferous aphids on host plants, which might lead to the dispersion of viruliferous aphids, thereby increasing the spread of PYV [53]. In our study, the phloem sap ingestion period of ToCV&TYLCV-viruliferous whiteflies was significantly shorter than that of ToCV-viruliferous whiteflies, indicating that the co-infection of ToCV and TYLCV could be beneficial for the transmission of both viruses. And it may explain why the phenomenon of plant virus co-infection is so universal in the field. Furthermore, it was shown that barley yellow striate mosaic virus (BYSMV) could infect the central nervous system of its vector *Laodelphax striatellus* and induce a prolonged phloem feeding period, which well confirmed the direct modification effect on the feeding behavior of vector insects brough by plant viruses [54].

## 5. Conclusions

This study systematically dissected the dual modulation of *B. tabaci* MED feeding behaviors under ToCV single-infection and ToCV&TYLCV co-infection through plant-mediated (indirect) and viruliferous (direct) pathways. Behavioral aversion was observed in whiteflies exposed to infected plants, as evidenced by prolonged non-probing durations and reduced phloem ingestion efficiency, suggesting host quality deterioration drives vector preference for healthy plants. Crucially, co-infected whiteflies exhibited accelerated phloem access kinetics, achieving the first E1 phase faster than non-viruliferous counterparts, a behavioral shift that aligns with enhanced viral transmission efficiency. These differential effects—suppression via host manipulation and stimulation through direct viral interaction—collectively elucidate the epidemiological linkage between *B. tabaci* MED performance decline and ToCV&TYLCV outbreak patterns in field conditions.

## Figures and Tables

**Table 1:** Statistical analysis of EPG parameters of *Bemisia tabaci* MED in this study.

Parameters	*p* Value ^a^	
	Tomato Plants ^b^	*Bemisia tabaci* MED ^c^
Non-phloem parameters		
1. Time to 1st probe from start	0.686	0.152
2. Total number of probes	0.592	0.183
3. Total duration of probes	0.646	0.064
4. Mean duration of probe	0.467	0.201
5. Total duration of np	**0.035**	0.086
6. Total duration of C	0.644	**0.016**
7. Duration of 1st probe	0.071	**0.003**
8. Time from 1st probe to 1st E	0.574	**0.014**
9. Number of probes before 1st E	0.477	0.594
10. Time from the beginning of that probe to 1st E	0.192	0.396
11. Total number of pd	**0.022**	0.093
12. Total duration of pd	**0.023**	0.302
Phloem parameters		
13. Total number of E1	0.350	0.657
14. Total duration of E1	0.200	0.979
15. Mean duration of E1	0.242	0.827
16. Total number of E2	0.350	0.582
17. Total duration of E2	**0.003**	**0.028**
18. Mean duration of E2	**0.016**	0.142
19. Percentage of probes reaching phloem phase	0.203	0.452
20. Percentage of phloem phases reaching waveform E2	0.117	0.307

^a^ *p* values were calculated using one-way ANOVA for normal distribution parameters, and Kruskal–Wallis H test for non-normal distribution parameters. Bolded *p* values are significant at *p* ≤ 0.05. ^b^ Non-viruliferous *B. tabaci* feeding on ToCV-infected, ToCV&TYLCV co-infected, and un-infected tomato plants. ^c^ ToCV-viruliferous, ToCV&TYLCV-viruliferous, and non-viruliferous *B. tabaci* feeding on cotton plants.

## Data Availability

The original contributions presented in this study are included in the article/Supplementary Materials. Further inquiries can be directed to the corresponding authors.

## Supplementary Materials

The following supporting information can be downloaded at https://www.mdpi.com/article/10.3390/insects16111091/s1: Table S1: Detection primers used in this study.
