# Supplementary material for: Distinct Modulation of Feeding Behavior in the Whitefly Vector Bemisia tabaci MED by ToCV Single-Infection Versus Synergistic Co-Infection with TYLCV
Source: Insects. 2025 Oct 24;16(11):1091. doi: 10.3390/insects16111091 (PMC12653564; doi:10.3390/insects16111091)
Supplement: Supplementary file 1 [file insects-16-01091-s001.zip › insects-3680653-supplementary.pdf]

Supplementary Table S1. Detection primers used in this study

Primer name	Primer sequence (5′-3′)
mtCOI-F	TTGATTTTTTGGTCATCCAGAAGT
mtCOI-R	CTGAATATCGRCGAGGCATTCC
ToCV-F	GGTTTGGATTTTGGTACTACATTCAGT
ToCV-R	AAACTGCCTGCATGAAAAGTC
TYLCV-F	ACGCATGCCTCTAATCCAGTGTA
TYLCV-R	CCAATAAGGCGTAAGCGTGTAGAC
